# Targeted Inactivation of Cerberus Like-2 Leads to Left Ventricular Cardiac Hyperplasia and Systolic Dysfunction in the Mouse

**DOI:** 10.1371/journal.pone.0102716

**Published:** 2014-07-17

**Authors:** Ana Carolina Araújo, Sara Marques, José António Belo

**Affiliations:** 1 Laboratory of Embryology and Genetic Manipulation, Regenerative Medicine Program, Departamento de Ciências Biomédicas e Medicina, Universidade do Algarve, Campus de Gambelas, Faro, Portugal; 2 IBB - Institute for Biotechnology and Bioengineering, Centro de Biomedicina Molecular e Estrutural, Universidade do Algarve, Campus de Gambelas, Faro, Portugal; 3 PhD Program in Biomedical Sciences, Universidade do Algarve, Campus de Gambelas, Faro, Portugal; 4 CEDOC – Chronic Diseases Research Center, Faculdade de Ciências Médicas, Universidade Nova de Lisboa, Lisboa, Portugal; Rutgers University -New Jersey Medical School, United States of America

## Abstract

Previous analysis of the *Cerberus like 2* knockout (*Cerl2^−/−^*) mouse revealed a significant mortality during the first day after birth, mostly due to cardiac defects apparently associated with randomization of the left-right axis. We have however, identified *Cerl2*-associated cardiac defects, particularly a large increase in the left ventricular myocardial wall in neonates that cannot be explained by laterality abnormalities. Therefore, in order to access the endogenous role of Cerl2 in cardiogenesis, we analyzed the embryonic and neonatal hearts of *Cerl2* null mutants that did not display a laterality phenotype. Neonatal mutants obtained from the compound mouse line *Cer2^−/−^::Mlc1v-nLacZ24^+^,* in which the pulmonary ventricle is genetically marked, revealed a massive enlargement of the ventricular myocardium in animals without laterality defects. Echocardiography analysis in *Cerl2^−/−^* neonates showed a left ventricular systolic dysfunction that is incompatible with a long lifespan. We uncovered that the increased ventricular muscle observed in *Cerl2^−/−^* mice is caused by a high cardiomyocyte mitotic index in the compact myocardium which is mainly associated with increased *Ccnd1* expression levels in the left ventricle at embryonic day (E) 13. Interestingly, at this stage we found augmented left ventricular expression of *Cerl2* levels when compared with the right ventricle, which may elucidate the regionalized contribution of Cerl2 to the left ventricular muscle formation. Importantly, we observed an increase of phosphorylated Smad2 (pSmad2) levels in embryonic (E13) and neonatal hearts indicating a prolonged TGFβs/Nodal-signaling activation. Concomitantly, we detected an increase of *Baf60c* levels, but only in *Cerl2^−/−^* embryonic hearts. These results indicate that independently of its well-known role in left-right axis establishment Cerl2 plays an important role during heart development in the mouse, mediating Baf60c levels by exerting an important control of the TGFβs/Nodal-signaling pathway.

## Introduction

The heart is the first organ to be formed to allow the efficient supply of the increasing nutritional requirements of the growing embryo [1]. A series of processes orchestrated by a complex genetic network and interplay of the diverse cardiac cell lineages is essential for a successful cardiogenesis [2]. Subtle perturbations during heart formation usually lead to congenital heart defects (CHD) [3], which are the most common congenital malformations worldwide [4].

In mice, the heart starts to be formed at gastrulation with the formation of the cardiac crescent at the anterior side of the embryo [5], which contributes to the heart primordium or first heart field (FHF) [6]. Cells from FHF will mainly give rise to the left ventricle (LV) [1]. Later, another region can be identified, the secondary heart field (SHF) that will mainly contribute to the right ventricle (RV) and outflow tract (OFT) [7]. The heart primordium region fuses at the embryonic midline to form a primitive heart tube [8]. In this primitive tubular phase, the heart loops to the right side of the embryo under the control of the signals that regulate left–right axis (L/R) [9]. After cardiac looping, two myocardial layers compose the primitive heart. The trabecular layer is a bundle of cardiomyocytes outlined by endocardial cells that project across the lumen of the ventricular chamber [10], and the compact layer is an organized multilayer that comprises the outmost ventricular region [11]. The cardiomyocytes that compose the compact layer have high proliferative and low differentiation capacities and the reverse is found in trabeculae. As development proceeds, the heart expands towards a four-chambered organ and the atrio-ventricular septation is established simultaneously with the correct alignment between arteries and their respective ventricles. This allows the development of the conducting and circulatory systems [12]. At the cellular level, the cardiomyocytes proliferate regulated by cyclins and cyclin-dependent kinase (CDKs) [13], [14] reaching two distinct high rates of DNA synthesis. The first occurs around midgestation (E12.5) and is associated with increased cardiomyocyte proliferation [15]. Later, in the first days after birth (P3–P4), a second peak of DNA synthesis is observed which ultimately results in binucleated cardiomyocytes [16]. Nonetheless, recent studies point to continued DNA synthesis and therefore to neomyocardialization potential in adult hearts [17], [18]. On the other hand cardiomyocyte differentiation occurs early in heart morphogenesis and persists until the first weeks of birth [19]. Thus the balance between cellular proliferation and differentiation during heart formation is crucial to provide the progressive thickening and maturation of the cardiac myoarchitecture [20].

*Cerberus like 2* (*Cerl2*) is a member of the Cer/Dan family, and has been shown to antagonize signals from the Transforming Growth Factor (TGF) type β superfamily [21]. The secreted protein Cerl2 binds to Nodal and contributes to asymmetric initiation of the left-right (L/R) axis [21], [22]. Accordingly, *Cerl2* knockout (*Cerl2^−/−^*) mice display L/R axis randomized and a significant mortality rate within a few hours after birth, mostly due to cardiac defects [21]. In addition, it has been reported that animals with laterality defects (LD) frequently have impaired cardiac function correlated with cardiac malformations [23], and a high mortality rate in mouse and humans [24], [25].

In this study we investigated the consequences of *Cerl2* loss-of-function in heart development, independent of the influence of LD on cardiac structure and function. We analyzed exclusively animals that did not show LD. Besides, emerging data has elucidated the role of *Cerl-1*, another member of Cer/Dan family, for cardiogenesis initiation, as reported in Xenopus [26], chicken [27] and in mouse embryonic stem cells [28], [29].

Here, we demonstrate that enlargement of the ventricular myocardial walls in *Cerl2* null mutants without LD is caused by cardiomyocyte hyperplasia possibly due to increased expression levels of *Ccnd1* at midgestation. Moreover, these animals showed impaired expression of cardiac genes during heart formation and reduced systolic function in early neonatal life. We also described that *Cerl2* expression levels are augmented in the LV at E13.5, indicating a possible preponderant function of Cerl2 in this ventricle during cardiogenesis. In accordance with these observations, we detected in *Cerl2^−/−^* embryonic hearts an increase of phosphorylated Smad2 (pSmad2) levels, a mediator of TGFβs/Nodal-signaling and of Baf60c levels, a subunit of SWI/SNF chromatin remodeling complex. Taken together, we conclude that Cerl2 emerges as an essential factor in the control of the TGFβs/Nodal-signaling acting as a modulator of the SWI/SNF-like BAF chromatin remodeling complex that takes place during embryonic cardiogenesis being this role essential for proper heart formation.

## Methods

### Ethics Statement

The studies involving animal experiments are in accordance to the ethical issues for clinical research and EU guidelines for animal research. All animal work performed in this study was conducted in compliance with the Portuguese law and approved by the Consultive Commission of the Veterinary Agency from Portuguese Ministry of Agriculture (Directive 2010/63/EU of the European Parliament), the Agency responsible for issuing approval for experimental animal studies, following the EU guidelines for animal research and welfare.

### Mice

The mouse lines used in this work, wild type, *Mlc1v-nLacZ24* and *Cerl2^−/−^* (129Sv background) were maintained at 20°C ±2°C in a 12 hour light-dark cycle. Noon of the day of detection of the vaginal plug was considered embryonic stage E0.5. Pregnant females were euthanized by cervical dislocation. The uterine horns were immediately removed and the embryos were dissected. After the echocardiography recordings, the deeply anesthetized neonatal mice (4% isoflurane mixed with 1L/minutes 100% Oxygen) were sacrificed by injection in the LV with a cardioplegic solution (1.5% KCl) to induce cardiac arrest.

### Sample Preparation

For paraffin and frozen tissue embedding the embryos and neonatal hearts were fixed in 4% paraformaldehyde (PFA) overnight (O/N). For qRT-PCR and Western Blot the whole embryos and isolated hearts were frozen directly in dry ice and stored at −80°C.

### Histology

The hearts were sectioned transversally in 10 and 5 µm thickness (embryos and neonatal hearts respectively). Haematoxylin and Eosin (H&E) and Masson-trichrome (TRI) staining were performed on paraffin-embedded sections, according to standard practices. To measure the wall thickness in embryonic hearts, three serial sections were chosen, having as guideline the four-chambers and the central conduction system [11]. Five parts per section were arbitrarily chosen for each mouse. The proportion of the compact and trabecular layers to the heart size was the ratio between their respective lengths and the longest diameter of the ventricle [30]. In neonatal hearts, five sections at the level of mid-papillary muscle were chosen to measure the anterior, lateral and posterior LV and RV walls, and the IVS as well. The data was normalized by the body weight. A blinded observer to mouse genotypes conducted the data analysis. AxioVision Image Software (Zeiss Company) was used for measurements.

### β-Galactosidase Staining

β-Galactosidase staining was performed in neonatal hearts according to standard procedures [31]. To perform morphometric analysis, the sections at mid-papillary muscle level were chosen and indexed to body weight.

### Immunohistochemistry

The immunohistochemistry (IHC) protocol was performed according standard procedures (Abcam protocol: http://www.abcam.com). Antibody sources are listed in Table S1.

### Cell counting and measurements

The quantification of proliferating cardiomyocytes in the compact layer of embryonic hearts was performed on 9 fields per ventricle (three fields per section) divided by the total of cardiomyocytes counted in each ventricle (n = 4–5 per genotype). The mitotic index to neonatal hearts was obtained from 9 fields of the right and left ventricular wall (AW, LW, and PW) and IVS divided by the total of cardiomyocytes counted per ventricular wall (n = 4–5 per genotype). The images were obtained using confocal microscopy (LSM 710, Zeiss Company; under 63X or 40X magnification for embryonic hearts, or fetal and neonatal hearts, respectively). Random regions of the compact layer were selected and fixed sizes of the fields were determined using Adobe Photoshop CS4 software to count the cardiomyocytes. The guidelines used to choose the sections were the same used to measure the myocardial thickness that were described in the Histology section of the methods.

The cardiomyocyte area was assessed from the LVAW, LVPW and IVS (6–8 sections per heart, n = 3), using optical sectioning with an Apotome microscope (Axio Imager Z2 Fluorescence, Zeiss Company; 63X magnification). We obtained the relative cell area through delineation of 100 cardiomyocytes for each mouse using Zen software (Zeiss Company).

The quantification of pSmad2 immunofluorescence signal in the cardiomyocytes present in the compact layer at E13 was conducted using ImageJ software and adapted from Dr. T. Nakamura Lab. protocol [32]. Briefly, we randomly selected nine fields per each ventricle using three sections per sample. In each image, the background fluorescence level was assessed by averaging background signals and was subtracted from the fluorescence in compact myocardium. Six randomly chosen fluorescent cardiomyocyte nuclei were marked manually per field, and then fluorescence values were obtained automatically.

A single observer blinded to mouse genotypes performed data analysis.

### Western blot

For total protein extracts, embryonic and neonatal hearts were lysed using protein extraction buffer (10 mM Tris HCl pH 7.5, 150 mM NaCl, 2 mM EDTA, 1% Nonidet P-40 and 10% Glycerol) supplemented with complete protease inhibitor cocktail (Roche) and a phosphatase inhibitor (Calbiochem®). Following extraction, total proteins were separated by SDS-PAGE polyacrylamide gel (8%), transferred onto Immun-Blot PVDF membrane (Biorad Laboratories), blotted with primary antibodies, and then incubated with horseradish peroxidase-conjugated secondary antibodies. Western blots (WB) were developed with Immun–Star WesternC Chemiluminescence Kit (Bio-Rad) and visualized using Quemi-Doc Imaging system (Bio-Rad). Densitometry measurements of relative protein quantities were determined by Image Lab 4.0 software (Bio-Rad). Antibody sources are listed in Table S1.

### RNA extraction and qRT-PCR

Total RNA was extracted from embryonic and neonatal hearts using TRI Reagent® (Sigma-Aldrich). RNA samples were treated with RNAse free DNase kit (Ambion) prior to reverse transcription. RNA quantity, quality and integrity were evaluated using a spectrophotometer (Nano Drop, Thermo Scientific) and Experion™ (Bio-Rad). First strand cDNA was synthesized using 0,5 µg of RNA, according to the manufacturer’s guidelines. Briefly, RNA was primed with oligo(dT) and the first strand was synthesized using SuperscriptII (Invitrogen). Triplicate qRT-PCR reactions were performed. Negative controls to assess genomic contamination were achieved for each genotype, without reverse transcriptase. Non-template control (without RNA template) also was included in the reaction as a negative control, which resulted in no detectable amplification product. Relative quantification was performed according to the 2^−ΔΔCt^ method [33], and normalized to GAPDH and HPRT1 as reference genes. Primer sequences for qRT-PCR are available upon request.

### Echocardiography

Neonatal mice were submitted to high-resolution echocardiography VEVO 2010 (VisualSonics, Toronto, Canada) using a 40-MHz high-frequency linear transducer. Mice were anesthetized with 0.8–1% Isoflurane mixed with 1 L/min 100% Oxigen delivered by a facemask during the procedure, using the minimum concentration required to suppress spontaneous body movements. Prewarmed gel was used as an ultrasound-coupling medium. Body temperature and heart rate could not be monitored due to small size of the animals. Two dimensional (B-) mode and spectral Color Doppler recordings were saved for subsequent analysis. Each ultrasound exam took 5–7 minutes per mouse (from the onset of anesthesia to the end of echocardiography procedures). The measures were obtained from three and five successive cardiac cycles (B-mode and Color Doppler, respectively), and the data was averaged. A blinded observer conducted the echocardiographic measures.

The heart rate (HR) was determined from 4–5 averaged wavelengths from aortic flow. From spectral Color Doppler tracing we obtained the aortic and pulmonary peak velocities (suprasternal and parasternal long axis views, respectively). The LVM (mg) was calculated according the Area-Length (AL) formula:
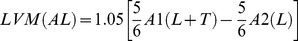



The factor 1.05 represents the myocardium density [34]; (A1) and (A2) means epicardial and endocardial border obtained through B-mode in parasternal short axis view (PSAX) at the end of systole; (L), the major length was obtained in parasternal long axis view (PLAX) from apex to aortic annulus, and (T) denotes the mean wall thickness calculated from (A1) and (A2).

The Stroke Volume (SV) is a product of the circular vessel area 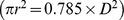 where (D) is a diameter of aortic annulus obtained from PLAX, and the time velocity integral (TVI) was obtained from aortic flow.
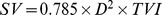



The Cardiac output (CO) is a product of the SV and the HR. The LVM index (mg/g) and the CO index (ml/min/g) were calculated by normalization with the body weight.

### Statistical Analysis

The data obtained from all analyses was statically analyzed using GraphPad PRISM 5 software. Statistical differences were determined by 2–tailed, unpaired Student *t* test. Probability values of *P*<0.05 were considered significant. All results are represented as mean ±SEM.

## Results and Discussion

*Cerl2^−/−^* neonates display left ventricular hypertrophy with increased left ventricular mass and severe cardiac dysfunction.

It has been extensively reported that laterality defects are characterized by failure in the L/R axis establishment followed by randomized positioning of the asymmetrical visceral organs [23]. In order to identify the animals without LD, we started our analysis by performing echocardiographic examination of *Cerl2^−/−^* neonatal hearts, and complemented it with observation of the relative positions of the heart and arteries, lung lobulation, liver and stomach disposition within the body. In contrast to the wild type (WT) controls, a third of *Cerl2^−/−^* neonates die immediately after birth and a few more until the time of weaning (data not shown). From the 52 living neonates analyzed, 38 (73%) did not display LD. These hearts were sectioned to further histomorphometry of the anterior (AW), lateral (LW) and posterior wall (PW) of the LV and RV. The interventricular septum (IVS) was also measured.

**Figure 1:**
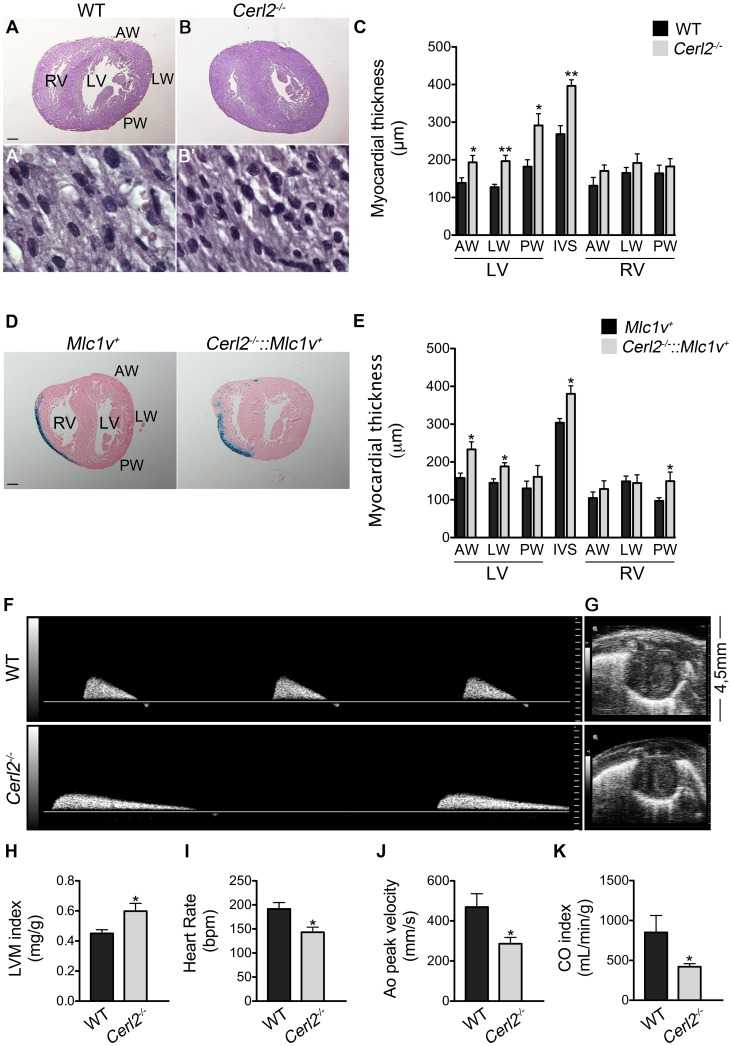
Increased compact myocardium and systolic dysfunction in *Cerl2^−/−^* neonates is independent of the LD phenotype. (A, B and D) Transverse sections (5 µm) of hearts stained with H&E (A and B) and β-Galactosidase staining counter-stained with Eosin (D). (A′ and B′) Note the numbers of cardiomyocytes per field (100X). (C and E) Quantitative measures of myocardial thickness in the compact layer indicate increased LV and IVS thickness (n = 5 and n = 4, respectively). (F–K) Echocardiographic analysis (n = 5–6 for WT and n = 7 for *Cerl2^−/−^*). (F) Represents the Ao peak velocity by Colour Doppler. (G) Represents the PSAX in B-mode view. (H) LVM index (mg/g) is increased in null mutants; (I) Heart Rate (bpm, beats per minute), (J) Ao peak velocity (mm/s) and (K) CO index (ml/min/g) are reduced in *Cerl2^−/−^* neonates. Ao, aorta artery; AW, anterior wall; CO, cardiac output; IVS, interventricular septum; LW, lateral wall; LV, left ventricle; LVM, left ventricular mass; PW, posterior wall and RV, right ventricle. Scale bars: 200 µm. * *P*<0.05 and ** *P*<0.01.

Histological analysis showed an enlarged LV myocardium and IVS in the *Cerl2^−/−^* neonates (Fig. 1A and 1B). Additionally, at higher magnifications we observed an increased cardiomyocyte number as seen by higher numbers of nuclei by section (Fig. 1A′ and 1B′). Histomorphometric evaluation revealed an increased thickening of the walls that constitute the LV myocardium and the IVS (Fig. 1C). Despite no statistical significant differences, there was a tendency toward the increase of the RV wall thickness in *Cerl2* null mutants when compared to WT controls.

In order to validate that the increased LV and IVS myocardial walls found in *Cerl2^−/−^* neonates is not related with the cardiac-associated laterality phenotype, we crossed our null mutant mice with the *Mlc1v-nlacZ-24* transgenic mouse line. This transgenic line contains a reporter transgene that mimics *Fgf10* expression in the developing heart by expressing β-galactosidase in the RV and OFT [35]. The neonates obtained by crossing the compound *Cerl2^+/−^:: Mlc1v-nlacZ-24^+^* were analyzed by combining the analysis of visceral organ arrangement with ventricle location of β-galactosidase staining. By using this method we were able to identify the animals that did not display cardiac LD, that as shown in Figure 1D, have the RV marked by β-galactosidade staining as in transgenic *Mlc1v-nlacZ-24^+^*. We analyzed only the ventricle walls of *Cerl2^−/−^: Mlc1v-nlacZ-24^+^* animals without LD, which were about half of the living neonates (6/11). Histomorphometry analysis in the compound animals (Fig. 1E), confirmed that the LV (AW and LW) and IVS myocardium is hypertrophic in the *Cerl2* mutants in the absence of LD. Moreover, in these compound animals the RVPW was significantly and the RVAW was tendentiously increased when compared with the control. Altogether these data suggest that in absence of Cerl2 the neonates showed evident ventricular hypertrophy, and this is independent of the LD phenotype.

To complement the hypertrophic phenotype study, we evaluated the left ventricular mass (LVM) and the cardiac function through non-invasive transthoracic echocardiography in neonatal mice (Fig. 1F–K). According to our analysis the LVM index, a useful parameter to detect hypertrophy, is increased in *Cerl2^−/−^* neonatal hearts (Fig. 1H). The wet heart weight/body weight ratio, which is also indicative of hypertrophy, has been described to be imprecise in smaller mice [36]. This could account for the fact that we did not find significant differences in this parameter when comparing with WT (Table S2). We determined the heart rate (HR) using the time interval between four-five successive waveforms on Color Doppler mode tracings of the aortic (Ao) peak velocity. According to our data, *Cerl2^−/−^* neonatal mice have a decreased HR (Fig. 1I). Furthermore, the LV function was also affected as suggested by the decreased ascending aorta artery peak velocity in *Cerl2* null mutants (Fig. 1F and 1J). In addition, the pulmonary systolic performance was also analyzed by the pulmonary (PA) peak velocity [37], and no significant differences were found in *Cerl2^−/−^* neonates (Table S2). The cardiac output index (COi), a parameter which indicates the systolic function [34] was calculated to the LV and a reduced COi was found in *Cerl2* mutants (Fig. 1K). Although these results demonstrate that the *Cerl2* null neonates display abnormal cardiac physiology, the basis for systolic dysfunction in *Cerl2^−/−^* is unknown so far. However, these animals exhibit an interesting postnatal phenotype, which may suggest that in absence of Cerl2, the neonatal mutants do not properly respond to increased hemodynamic workload required to switch from pre- to postnatal phase. Taken together, the data presented here indicates that the LV hypertrophy observed in *Cerl2^−/−^* is associated with impaired left ventricular systolic function.

### The thickening of ventricular myocardial wall in *Cerl2^−/−^* mice starts to develop already during embryonic heart development

**Figure 2:**
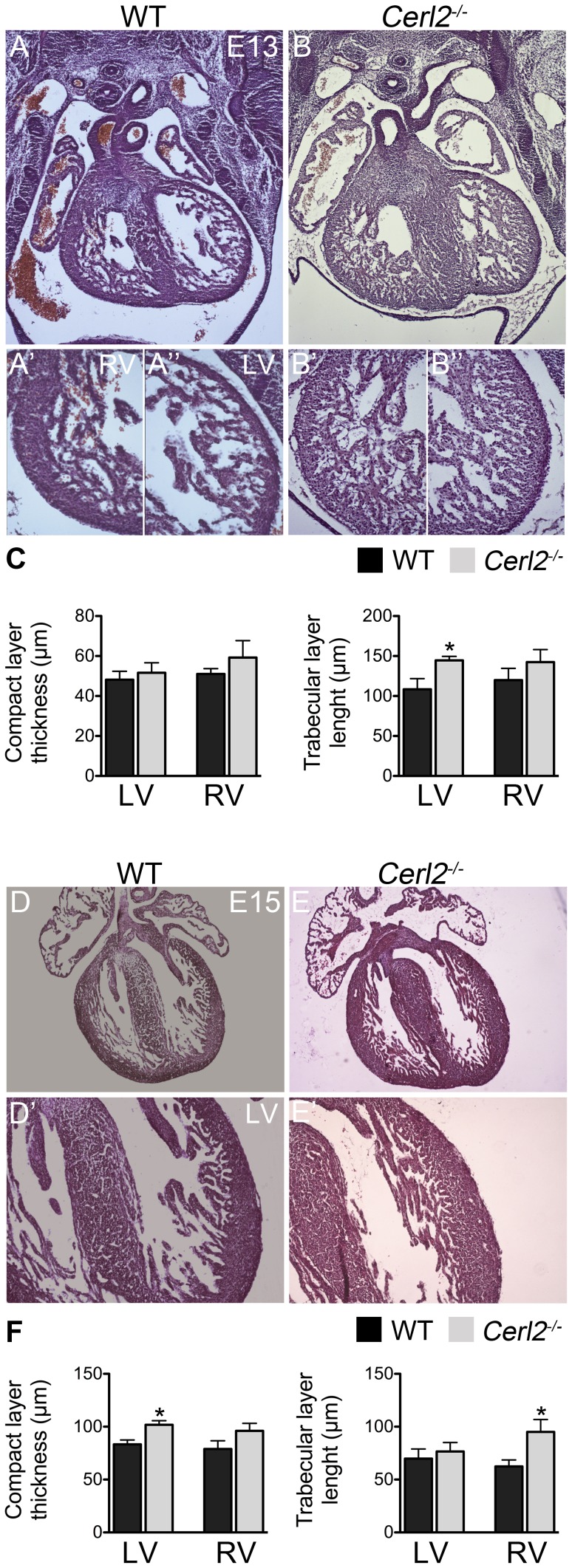
Progressive increase of compact myocardium is already detected in *Cerl2^−/−^* embryos. (A–B and D–E) Transverse sections (10 µm) stained with H&E at E13 and E15 respectively (5X). (C and F) Quantitative measurements of myocardial thickness in the compact and trabecular layers are depicted (E13, n = 4 and E15, n = 5). Note the progressive increase of the compact myocardium after the compaction process. LV, left ventricle and RV, right ventricle. A′–A′′ and B′–B′′; 20X, and D′ and E′, 10X. * *P*<0.05.

In order to understand if the increase of the LV walls and IVS found in neonatal hearts originates during prenatal stages, we analyzed the trabecular and compact layers in embryonic and fetal hearts, which are crucial to increased thickness of the heart during cardiogenesis. The trabeculae become compacted towards the compact layer around at E14 consequently at E16 onwards the myocardium is mainly constituted by compact layer [20], [38]. Therefore, we decided to investigate the myocardial growth before and after the compaction process (E13.25 and E15.25, respectively). According to a previous report, the RV and LV show no differences in the myocardial thickness before this process [20] and, histomorphometric analysis showed that at E13–13.25, the compact layer in both ventricles of *Cerl2^−/−^* mutant also seems not to differ from WT (Fig. 2C). In contrast, the trabecular layer of the LV is larger in *Cerl2^−/−^* (Fig. 2A–A′′, B–B′′ and C), and despite the trend no statistical difference was found in the right ventricular trabecular layer when comparing with WT (Fig. 2A–A′, 2B–B′ and 2C). These results may indicate that increased LV trabecular expansion could lead to an increase of the compact layer during the compaction process in *Cerl2^−/−^* embryonic hearts.

At the second time point studied, at E15–15.25, *Cerl2^−/−^* hearts showed a thicker LV compact layer (Fig. 2D–D′, 2E–E′ and 2F). However, the LV trabecular length was now similar to WT. The thickness of the right ventricular compact layer of *Cerl2^−/−^* at this stage did not differ from WT. In contrast, *Cerl2^−/−^* showed seemingly an augmentation of the right ventricular trabeculation (Fig. 2F), however it was not sufficient to generate a statistically significant enlarged RV myocardium. The hypertrabeculation usually is associated with non-compaction in the ventricles, resulting in a thin compact layer [39], [40]. Despite the increased trabeculae expansion observed in the RV of *Cerl2^−/−^*, thinner RV myocardial walls were not observed. This shift from thickened LV trabecular layer at E13 in *Cerl2^−/−^* to thickened compact layer at E15 may be likely related with the compaction process.

### Mitotic cardiomyocytes are increased in compact myocardium of *Cerl2^−/−^* embryonic and neonatal hearts

To investigate the cellular phenotype underlying the hypertrophic cardiomyopathy, we evaluated the number of mitotic cardiomyocytes in *Cerl2^−/−^* embryos (E13–13.25), fetuses (E15–15.25) and neonates (P0), which during embryonic mouse development, are singly nucleated [41]. To that end, we immuno-labeled cardiomyocytes with heavy chain cardiac myosin (MF-20) or sarcomeric α-actinin antibodies and co-labeled with the mitotic marker phospho-Histone H3 (pH3) antibody. This allowed us to calculate the mitotic index as the ratio between proliferating cardiomyocytes and the total number of cardiomyocytes.

**Figure 3:**
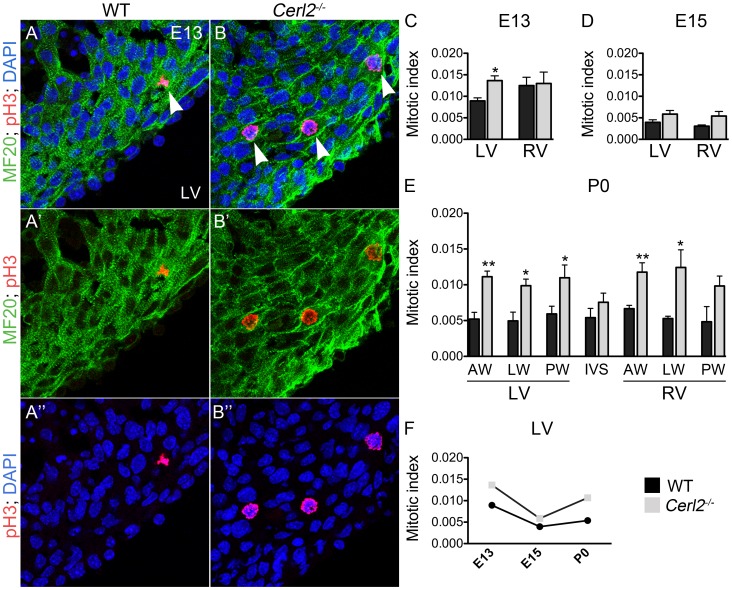
*Cerl2^−/−^* animals display increase in the ventricular mitotic index. (A and B) Corresponds to merged images at E13 of MF20 (green), pH3 (red) and DAPI (blue). (A′ and B′) MF20 and pH3; (A′′ and B′′) pH3 and DAPI (63X). White arrowheads indicate proliferating cardiomyocytes in the compact myocardium. (C–F) Proliferating cardiomyocytes observed in different stages of the heart development. (C) E13–13.25, n = 4; (D) E15–E15.25, n = 5; (E) P0 hearts, n = 5. (F) The timeline of mitotic cardiomyocytes in the LV. aw, anterior wall; IVS, interventricular septum; LW, lateral wall; LV, left ventricle; PW, posterior wall and RV, right ventricle. * *P*<0.05 and ** *P*<0.01.

At E12.5, the first peak of DNA synthesis is normally observed in cardiomyocytes [42]. At E13–13.25, we observed in the LV of the compact myocardium an elevated cardiomyocyte proliferation (Fig. 3A–A′′, Fig. 3B–B′′ and Fig. S3) and an increased mitotic index evidenced in *Cerl2^−/−^* embryos (Fig. 3C), being this result from the quantification of the proliferating cardiomyocytes present in the compact myocardium through 9 random fields/ventricle under 63X magnification (n = 4–5 per genotype). No difference in mitotic index was observed in the compact RV myocardium at this stage.

After the compaction process, a natural reduction of the cardiac cell cycle occurs [43]. In *Cerl2* null mutants at E15–15.25, no statistically significant difference was found in the compact myocardium in any of the ventricles, despite the tendency to maintain an increased mitotic index (Fig. 3D). Continuing the time course investigation on myocardial growth, we analyzed neonates within few hours of birth (P0) to ensure that we would evaluate essentially single nucleated cells, before the second peak of DNA synthesis that leads to the formation of bi-nucleated ventricular cardiomyocytes (P3–P4) [44]. We sub-divided the myocardium into AW, LW and PW to define the pattern of proliferation in each ventricular wall. The results showed a significantly higher proliferation index in all LV walls of *Cerl2^−/−^* (Fig. 3E). Unexpectedly, we found a high variability of the mitotic index in the IVS. According to literature, the proliferation capacity of the IVS decreases upon the completion of the septation process and, from midgestation onwards, it is mostly the LV myocardial population that contributes to IVS formation [45]. Therefore, it is likely that the increased thickness found in IVS in *Cerl2* mutants is due to a substantial influence from the LV. Although we observed an increased mitotic index in the RVAW and RVLW (Fig. 3E), this event was not able to induce marked increased on myocardial thickness. The distinct cell-lineage sources, roles and workloads between the ventricles may explain the intrinsic capacity of the LV to be enlarged [46].

To investigate whether the increased mitosis occurs in response to stimuli from increased cardiac cell death, we observed the apoptotic cells in *Cerl2^−/−^* neonatal mice using the anti-cleaved Caspase-3 in order to compare the levels of apoptosis in *Cerl2^−/−^* neonatal hearts with WT. We did not find alterations in any sub-region of the LV or IVS (data not shown). These results indicate that the general increase of the mitotic index observed in the compact layer of the *Cerl2^−/−^* mutant is not related with alterations in the levels of cellular death.

Besides the hyperplasic cardiac phenotype found in *Cerl2^−/−^* neonates, we also investigated whether hypertrophy was also involved in thickened LV walls and IVS. For that we evaluated the relative cardiomyocyte area of the AW and PW of the LV and of the IVS through co-immunostaining using MF20 and laminin as markers of cardiomyocyte and cardiomyocyte membranes, respectively (Fig. S1). Our analysis revealed no differences between the genotypes, suggesting that *Cerl2^−/−^* neonatal hearts do not display hypertrophy at the cellular level. Additionally, cardiac fibrosis is a classical feature of hypertrophy and cell death [47] and no evidence of fibrosis in *Cerl2^−/−^* neonatal hearts was revealed through Masson Trichrome staining (Fig. S2). Collectively, these data demonstrates that in *Cerl2^−/−^* the increased myocardial thickness seen mainly in the LV is caused by cardiomyocyte hyperplasia and not by hypertrophy (Fig. 3F).

### The expression of cardiac genes is affected in *Cerl2^−/−^* mice

**Figure 4:**
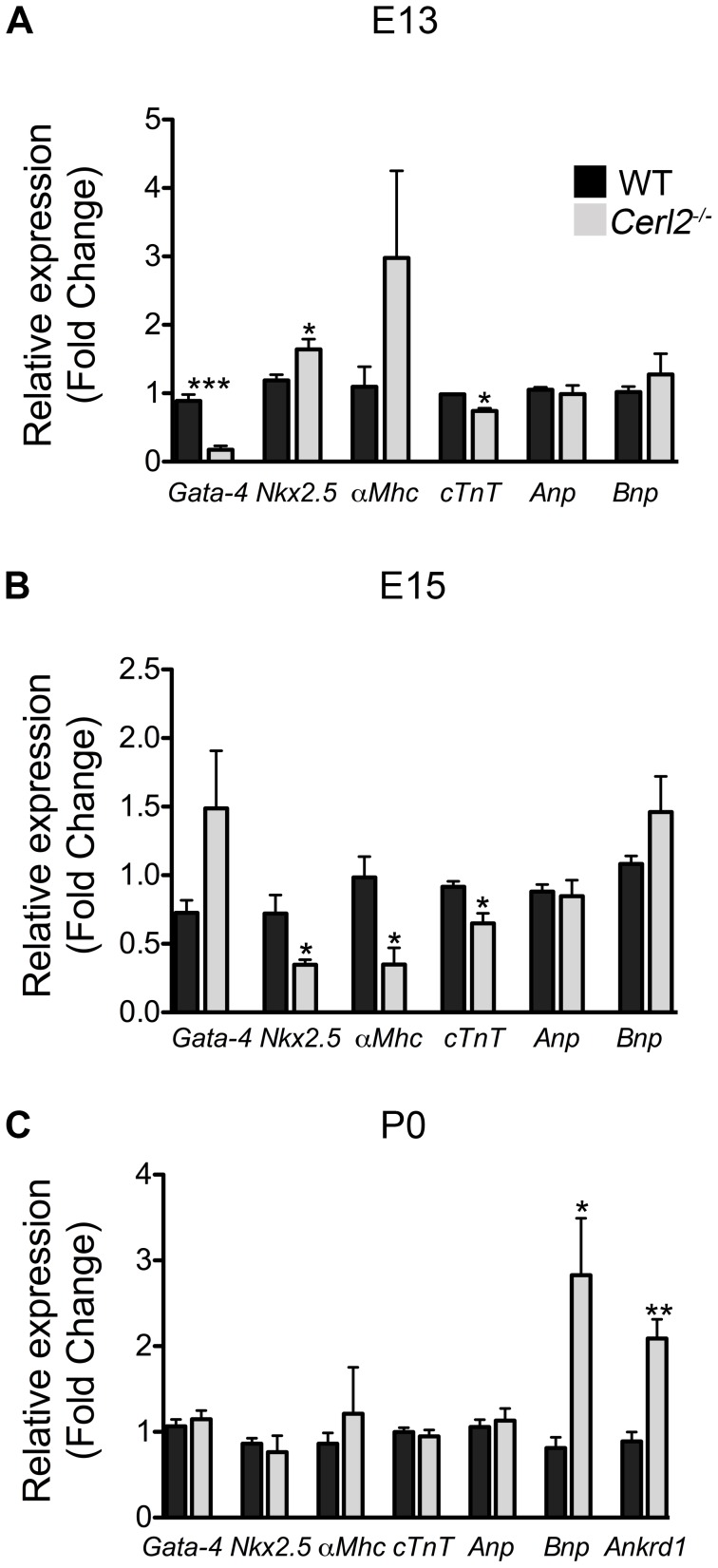
Relative mRNA expression levels of cardiac genes is altered in *Cerl2^−/−^* hearts. qRT-PCR was performed in triplicate for *Gata-4*, *Nkx2.5*, *α-Mhc*, *c-Tnt*, *Anp, Bnp* and *Ankrd1*. Note the altered expression of cardiac genes during the development in absence of Cerl2 (A) E13, n = 4. (B) E15–15.25, n = 4 and (C) P0, n = 5. * *P*<0.05, ** *P*<0.01 and *** *P*<0.001.

To evaluate whether the absence of *Cerl2* affects the expression level of cardiac genes from midgestation onward, we performed qRT-PCR analysis of transcripts isolated from hearts at E13–E13.25, E15–E15.25 and P0. We analyzed the expression of transcription factors that are important for normal heart development such as *Gata-4* and *Nkx2.5* [3]. We also analyzed structural genes involved in contractility like *α-Mhc* and *cTnT*, which are activated by the transcription factors *Gata-4* and *Mef2* [48]. In addition, genes such as *Anp*, *Bnp* and *Ankrd1,* which are known to be involved in the hypertrophy program and cardiac stress [49], [50] were evaluated. qRT-PCR analysis showed a dramatic reduction of *Gata-4* expression and a slight decrease of *c-TNT* in *Cer-2^−/−^* hearts at E13 (Fig. 4A). In contrast, we detected an increase in *Nkx2.5* expression levels, and at E15 we observed a decreased *Nkx2.5* expression level. Since alterations in this gene are associated with conduction abnormalities [51], we speculate that *Cerl2^−/−^* may have impaired cardiac function already during fetal development. Concurrently, we also detected reduction of the encoding contractile genes *α-Mhc* and *cTnT* but not altered *Gata-4* in *Cerl2^−/−^* (Fig. 4B) at this stage. According with other studies, the alterations of transcription factors such as *Gata-4, Nkx2-5* and *Mef2* and their target genes may compromise the cardiomyocyte differentiation program [48], [52]. Therefore, we believed that the cardiac function of the null mutants might already be affected during embryogenesis. However, at E13 and E15 we did not detect any alteration of *Anp* and *Bnp* expression in *Cerl2^−/−^* mutants, which indicates that the blood pressure and blood volume regulating the hypertrophic response in embryonic stages are not de-regulated [49].

In *Cerl2^−/−^* neonatal hearts, we have however detected a substantial increase of *Bnp* and *Ankrd1* expression (Fig. 4C), suggesting early cardiac stress in neonates. It has been recently demonstrated that *Ankrd1* is involved in the proliferation of cardiomyocytes and cardiomyopathy in humans [50], [53]. Therefore, the increased *Ankrd1* expression may be associated with the ventricular hyperplasia found in *Cerl2^−/−^* neonates. However, no significant alteration was found for other cardiac genes (*Gata-4*, *Nkx2.5*, *α-Mhc*, *c-Tnt* and *ANP*) at this stage. Collectively, the observed changes in the expression levels of essential genes for cardiogenesis indicate a de-regulation of the cardiac genetic program in *Cerl2* mutant embryos, which consequently compromises the cardiac function and survival of these animals.

### The regulatory role of Cerl2 in the cardiomyocyte cell cycle machinery leads to increased expression of *Cyclin type D1* in *Cerl2^−/−^* embryonic hearts

**Figure 5:**
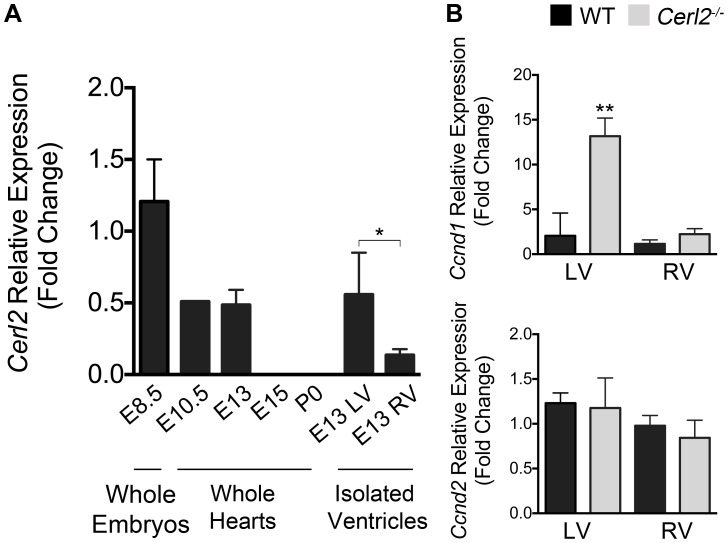
The increased expression level of *Ccnd1* suggests the regulatory role of Cerl2 during cardiogenesis. (A) mRNA Cerl2 levels were normalized to reference genes and compared with WT expression at E8.25–8.5. At E8.25–8.5, pool of two litters (whole embryos, n = 6 each); E10.5, pool of one litter (isolated hearts n = 6); E13, E15 and P0 isolated hearts (n = 5); E13 pool of two microdissected left and right ventricles (n = 3). The chart reveals the expression level of *Cerl2* until midgestation and in isolated ventricles, *Cerl2* is more expressed in the LV. (B) *Ccnd1* and *Ccnd2* relative expression level in isolated ventricles at E13. qRT-PCR was performed in triplicate. LV and RV indicate left and right ventricles, respectively. * *P*<0.05 and ** *P*<0.01.

Previous work have shown the expression of *Cerl2* in the mouse node and its function in the initiation of the symmetry breaking [21], [22]. To combine the cardiac phenotype in *Cerl2* null mutants with the putative cardiac function of Cerl2, we evaluated its expression level during cardiac formation through qRT-PCR analysis using isolated embryonic, fetal and neonatal mouse hearts. We used WT embryos at node stage (E8.25–8.5) and *Cerl2^−/−^* embryonic hearts as positive and negative controls, respectively. Relative *Cerl2* mRNA expression was found in isolated hearts at E10.5 and E13 although seemingly lower than the expression in node stage embryos (Fig. 5A). In addition, we could not detect *Cerl2* expression in the heart beyond midgestation stages (E13). We then microdissected the LV and RV at E13 in order to assess the levels of *Cerl2* expression in each ventricle, and we have found more expression in the LV than in the RV (Fig. 5A). As expected, we did not detect *Cerl2* expression in *Cerl2^−/−^* hearts (data not shown). These results suggest that *Cerl2* might have an important role during early cardiogenesis, mainly in the LV myocardium formation.

In order to investigate the master regulators of G1/S phase [13], [16], we quantified the relative mRNA expression of type *D Cyclins* (*Ccnd1* and *Ccnd2*) in isolated ventricles. Despite the similar expression levels of *Ccnd2* in both genotypes, our data uncovered an increase of *Ccnd1* mainly in the LV of *Cerl2^−/−^* at E13 (Fig. 5B) suggesting that the cardiac hyperplasia in the embryonic mutant hearts is due to overexpression of *Ccnd1* in the LV. These results are consistent with the cardiac phenotype found in *Cerl2^−/−^*. Moreover, we conclude that Cerl2 may play a specific mediator role during cardiomyogenesis, and therefore in its absence, the marked ventricular hyperplasia is observed predominantly in the LV.

### Absence of *Cerl2* leads to increased phosphorylated Smad2 signaling and up-regulation of Baf60c in embryonic hearts

**Figure 6:**
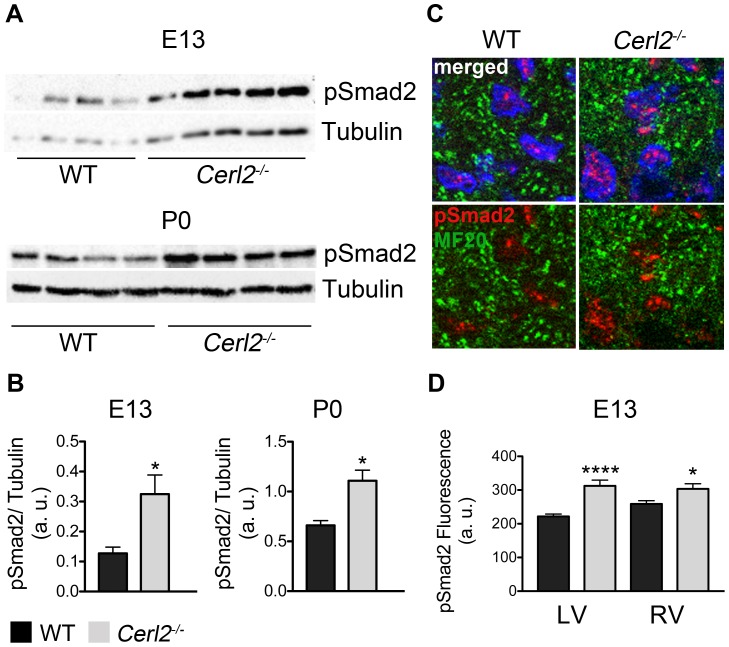
pSmad2 levels are increased in *Cerl2^−/−^* embryonic and neonatal hearts. (A) Western blot at E13 (WT, n = 4 and *Cerl2^−/−^* n = 5, upper) and P0 (n = 4, bottom). (B) Quantitative analysis of anti-pSmad2 normalized with anti-Tubulin at E13 on the left and P0 on the right, respectively. (C) Immuno-labeled of pSmad2 in left ventricular compact layer at E13. The cardiomyocyte cells (green, labeled with MF20) were co-labeled with phosphorylated Smad2 (red). The nuclei were stained with DAPI (100X). (D) Quantitative analysis of pSmad2 immuno-labeled in the LV and RV (arbitrary units) (n = 3). a. u., arbitrary units; LV and RV indicate left and right ventricles, respectively. * *P*<0.05 and **** *P*<0.0001.

Binding of TGFβs/Nodal/Activin to its receptors leads to phosphorylation of the intracellular proteins known as receptor-regulated Smads (Smad2 and Smad3) [59]. Then the phosphorylated (p) Smad2/3 interact with the co-factor Smad4 forming a transcriptional complex, which will translocate to the nucleus to regulate the downstream TGFβs/Nodal target genes [60]. Since Cerl2 is a TGFβs/Nodal antagonist, we postulated that the absence of *Cerl2* might cause alteration in levels of TGFβs/Nodal-signaling. Therefore, we evaluated the phosphorylation status of Smad2 (pSmad2) in protein extracts from embryonic (E13) and neonatal (P0) hearts. The quantification of pSmad2 by Western blot revealed increased pSmad2 in *Cerl2^−/−^* embryonic (E13) (Fig. 6A, upper and 6B, left) and neonatal hearts (Fig. 6A, bottom and 6B, right), suggesting an increased transcriptional activity of TGFβs/Nodal-signaling. Here, we propose two hypotheses that might explain the elevated phosphorylation of Smad2 found in *Cerl2^−/−^* neonatal hearts. First, the autoregulatory loops are common in this type of signaling, making it possible that the absence of *Cerl2* at earlier stages allows the prolongation of TGFβs/Nodal-signaling until later stages; and second, Cerl2 may interact with other protein(s) that could extend that signaling activation in the early neonatal period.

We also quantified the level pSmad2 on each ventricular compact myocardium of the *Cerl2^−/−^* embryos at E13 by quantitative immunofluorescence analysis (Fig. 6C and 6D) and this analysis showed that the pSmad2 levels on both ventricles are higher than the control. Interestingly, the bimodal role of the TGFβs/Nodal-signaling has been reported in the regulation of cardiogenesis [61]. First, through mesodermal and endodermal induction to promote cardiac induction and later, to control cardiomyocyte differentiation [61], [62], [63].

**Figure 7:**
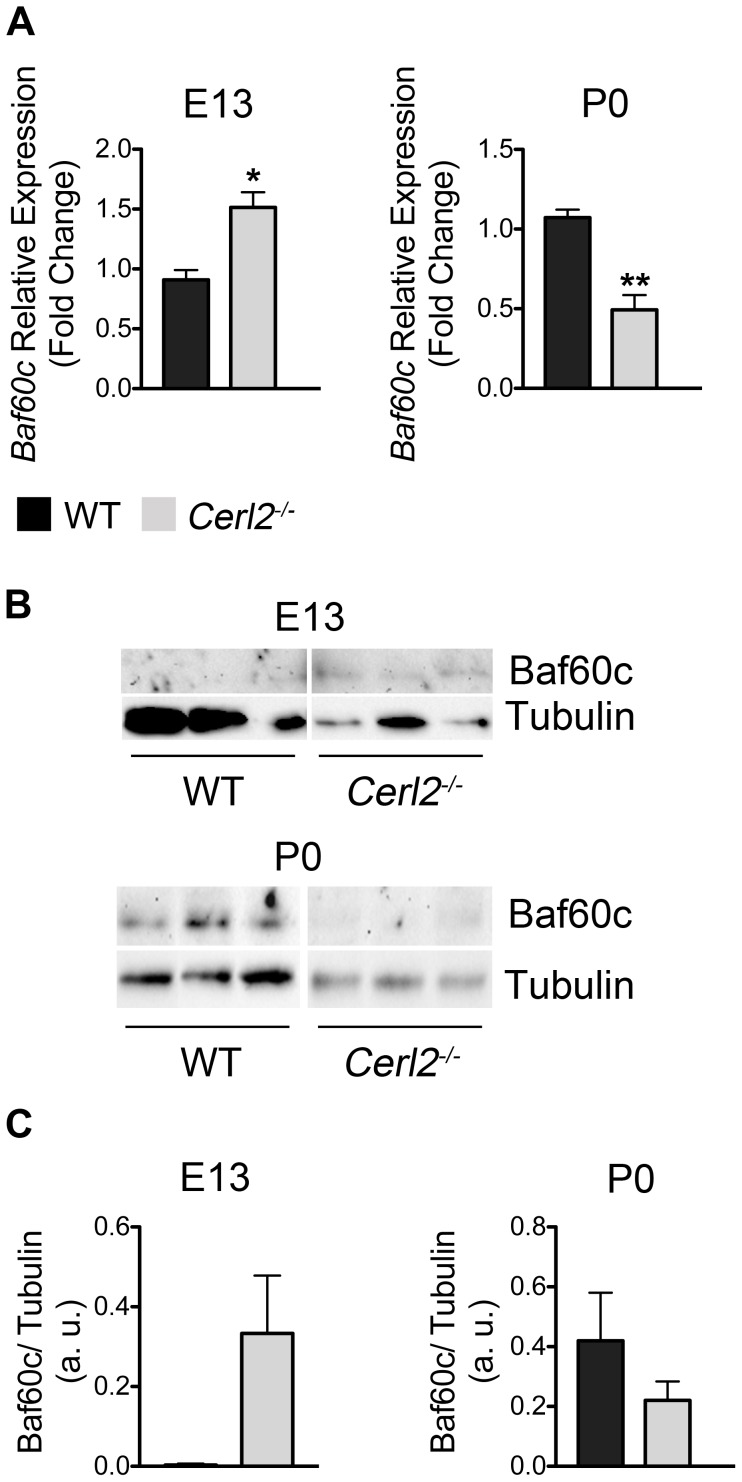
The absence of Cerl2 in embryonic hearts leads to up-regulation of Baf60c. (A) qRT-PCR was performed in triplicate for *Baf60c* at E13 (WT, n = 4 and *Cerl2^−/−^*, n = 3) and P0 (WT, n = 4 and *Cerl2^−/−^*, n = 5). (B) Western blot at E13 and P0 (n = 3) and (C) Quantitative analysis of anti-Baf60c normalized with anti-Tubulin. Note that no signal of Baf60c was detected in two out of three WT samples at E13, which precluded the statistical analysis. a. u., arbitrary units. * *P*<0.05 and ** *P*<0.01.

Work from various laboratories has demonstrated the role of the ATP-dependent SWItch/Sucrose NonFermentable (SWI/SNF) chromatin-remodeling complexes in modulating the transcription of target genes. The SWI/SNF complex is composed by different members such as Brahma related gene 1 (*Brg1*) or Brahma (*Brm*)-associated factors (BAF) [54]. One of them is *Smarcd3* that encodes the BAF subunit, Baf60c. This epigenetic gene has a fundamental role during cardiogenesis, acting as a mediator between SWI/SNF chromatin remodeling complex and cardiac transcription factors [55]. Moreover, Baf60c RNAi knockdown embryos present severe heart defects, reduced myocardial proliferation in the ventricles and altered expression of cardiac markers, causing lethality at E10–11 [56]. Interestingly, it was reported that another member of Cerberus family, Cerl1 acts as an early but not as a later cardiac inductor in *Xenopus* and in chicken [26], [27]. Moreover, Cerl1 has been described to be in the same regulatory network as Baf60c in mouse embryonic stem cells (mESC) cardiogenesis [28], [29]. All this prompted us to analyze whether the absence of Cerl2 signals alters the levels of Baf60c during cardiogenesis *in vivo*. We evaluated *Baf60c* mRNA and protein expression in whole hearts at E13 and P0 (Fig. 7A–C). Unexpectedly, we found increased mRNA and protein expression levels of Baf60c in *Cerl2^−/−^* hearts at E13. Since the SWI/SNF chromatin remodeling complex is involved in controlling cellular proliferation [57] and the overexpression of Baf60c stimulates the proliferation of neural progenitor cells [58], the increase of Baf60c expression seems to be consistent with the ventricular hyperplasia phenotype found in the *Cerl2* null mutants at E13. However in latter midgestation stages *Cerl2* was not detected and therefore in neonatal hearts inactivation of Cerl2 is not relevant to sustain the increased Baf60c levels. In conclusion, we postulate that the main involvement of Cerl2 in cardiomyocyte proliferation occurs during embryonic stages.

Nevertheless in mESC knockdown of Cer1 decreased Baf60c mRNA and protein levels, through regulation of TGFβs signals [28] very early in cardiogenesis, suggesting a different role for these two Cerberus molecules in cardiogenic events that may be related to their different domains of expression during heart development.

Although some detailed studies need to be addressed in order to access the interplay between Cerl2 and the SWI/SNF complex, our findings contribute to unveil the key role of Cerberus family during heart development in the mouse.

Here, we provide evidence that an increase in the TGFβs/Nodal-signaling levels may be responsible for the cardiac ventricular phenotype in *Cerl2^−/−^* hearts. Moreover, this increase of TGFβs/Nodal-signaling *in vivo* is associated with a concomitant increase in Baf60c levels.

## Conclusion

In conclusion, our study provides the first evidence of the role of Cerl2 during cardiac development independent of its function in establishment of the L/R asymmetry. This study reveals an important breakthrough in the function of Cerl2 to control growth factor activity in cardiogenesis. We demonstrate that absence of *Cerl2* leads to ectopic TGFβs/Nodal- signaling in the heart leading to a massive increase of cardiac walls possibly mediated by the SWI/SNF-like BAF complex. In the future, a tissue-specific cardiac deletion of *Cerl2* would be relevant to determine its precise contribution during the heart formation.

## Supporting Information

Figure S1 **Relative cardiomyocyte area in** ***Cerl2^−/−^*** **neonatal hearts.** (A) Represents the LVAW labeling with anti-laminin (*green*), 63X. (B) Relative cardiomyocyte area was measured in 100 cells (µm^2^), n = 3. LVAW, left ventricle anterior wall, LVPW, left ventricle posterior wall and IVS, interventricular septum. * *P*<0.05.
(TIF)

Figure S2 **No fibrosis was detected in** ***Cerl2^−/−^*** **neonates. Masson-**trichrome staining did not reveal fibrosis in *Cerl2* mutants (20X).
(TIF)

Figure S3 ***Cerl2^−/−^*** **animals display increased pH3 immunoreactivity in the left ventricle.** (A and B) Correspond to merged images at E13 of MF20 (green), pH3 (red) and DAPI (blue). (A′ and B′) MF20 and pH3; (A′′ and B′′) pH3 and DAPI. (A′′′ and B′′′) pH3. LV, left ventricle (10X). Scale bar: 100 µm.
(TIF)

Table S1 **Antibodies used in this study.**
(DOCX)

Table S2 **Echocardiographic parameters in** ***Cerl2^−/−^*** **neonates.**
(DOCX)
