# Supplementary figures and images for: Targeted Inactivation of Cerberus Like-2 Leads to Left Ventricular Cardiac Hyperplasia and Systolic Dysfunction in the Mouse
Source: PLoS One. 2014 Jul 17;9(7):e102716. doi: 10.1371/journal.pone.0102716 (PMC4102536; doi:10.1371/journal.pone.0102716)

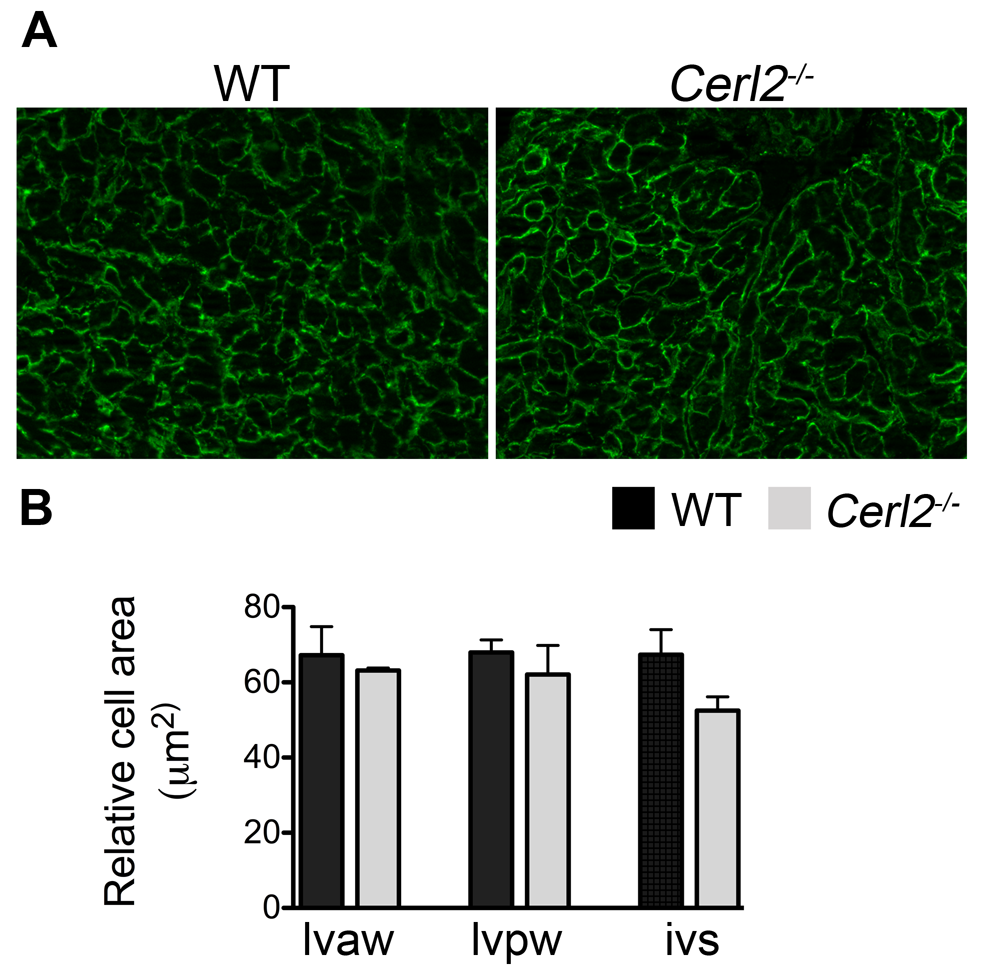

Supplement: Figure S1 — Relative cardiomyocyte area in Cerl2−/− neonatal hearts. (A) Represents the LVAW labeling with anti-laminin (green), 63X. (B) Relative cardiomyocyte area was measured in 100 cells (µm2), n = 3. LVAW, left ventricle anterior wall, LVPW, left ventricle posterior wall and IVS, interventricular septum. * P<0.05. (TIF) [file pone.0102716.s001.tif]

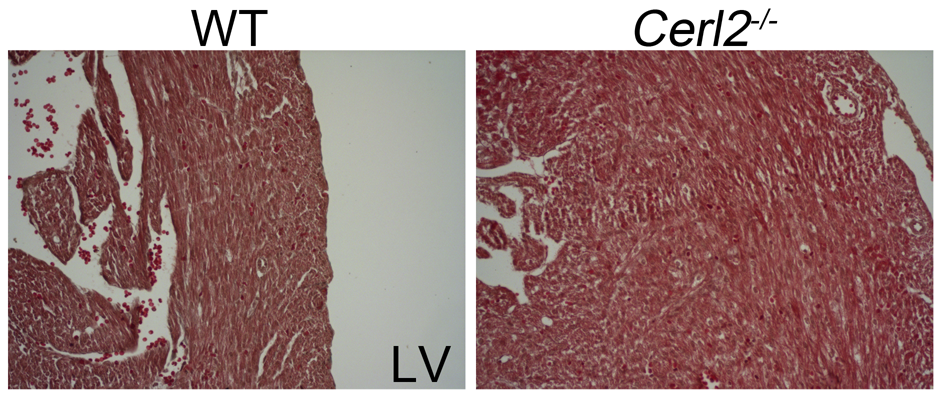

Supplement: Figure S2 — No fibrosis was detected in Cerl2−/− neonates. Masson-trichrome staining did not reveal fibrosis in Cerl2 mutants (20X). (TIF) [file pone.0102716.s002.tif]

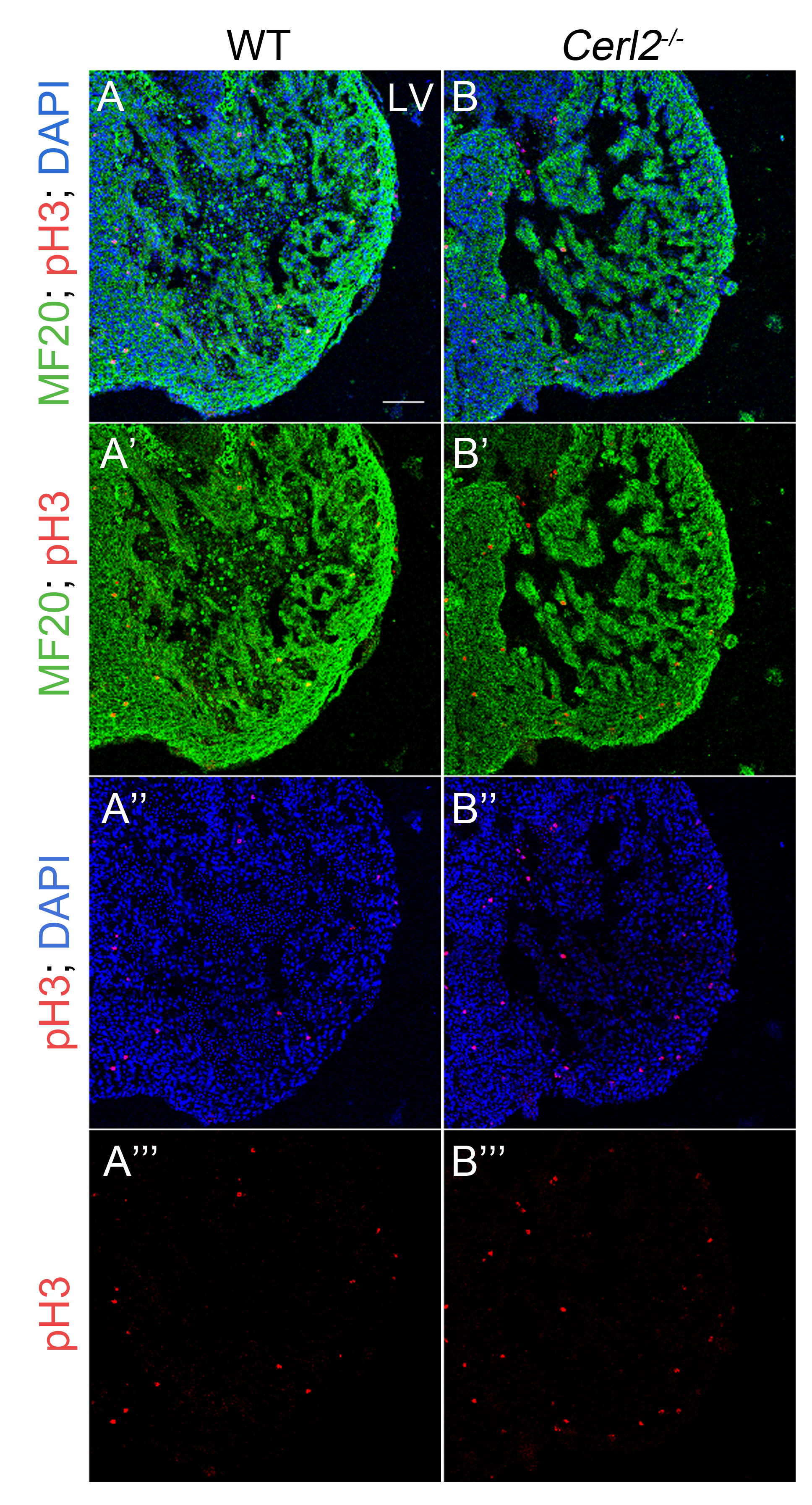

Supplement: Figure S3 — Cerl2−/− animals display increased pH3 immunoreactivity in the left ventricle. (A and B) Correspond to merged images at E13 of MF20 (green), pH3 (red) and DAPI (blue). (A′ and B′) MF20 and pH3; (A′′ and B′′) pH3 and DAPI. (A′′′ and B′′′) pH3. LV, left ventricle (10X). Scale bar: 100 µm. (TIF) [file pone.0102716.s003.tif]
