# Supplementary material for: Targeted Inactivation of Cerberus Like-2 Leads to Left Ventricular Cardiac Hyperplasia and Systolic Dysfunction in the Mouse
Source: PLoS One. 2014 Jul 17;9(7):e102716. doi: 10.1371/journal.pone.0102716 (PMC4102536; doi:10.1371/journal.pone.0102716)
Supplement: Table S1 — Antibodies used in this study. (DOCX) [file pone.0102716.s004.docx]

**Table S1. Antibodies used in this study**

| Aplication | Antibody | Dilution | Company |
| --- | --- | --- | --- |
| IHC | Laminin | 1:150 | Abcam, ab11575 |
| IHC | pH3 | 1:800 | Cell Signaling, #9701 |
| IHC | MF20 | 1:200 | Abcam, ab15 |
| IHC | α-Actinin Sarcomeric | 1:500 | Sigma, A7811 |
| IHC | Caspase-3 | 1:200 | R&D, AF835 |
| IHC | Goat anti-mouse | 1:1000 | Invitrogen, A21235 |
| IHC | Goat anti-rabbit | 1:1000 | Invitrogen, A11012 |
| WB | pSmad2 | 1:500 | Cell Signaling, #3101 |
| WB | α-Tubulin | 1:50000 | Sigma, T6199 |
| WB | Goat anti-mouse HRP | 1:25000 | Sigma, A4416 |
| WB | Goat anti-rabbit HRP | 1:25000 | Sigma, H6908 |

IHC, immunohistochemistry; WB, western blot
