# Supplementary material for: Targeted Inactivation of Cerberus Like-2 Leads to Left Ventricular Cardiac Hyperplasia and Systolic Dysfunction in the Mouse
Source: PLoS One. 2014 Jul 17;9(7):e102716. doi: 10.1371/journal.pone.0102716 (PMC4102536; doi:10.1371/journal.pone.0102716)
Supplement: Table S2 — Echocardiographic parameters in Cerl2−/− neonates. (DOCX) [file pone.0102716.s005.docx]

**Table S2. Echocardiographic parameters in *Cerl2^-/-^* neonates.**

| **Parameters** | **WT** | ***Cerl2^-/-^*** | ***P*** |
| --- | --- | --- | --- |
| Vent./Body | 5.27 ± 0.19 | 4.98 ± 0.3 | ns |
| Pa (mm/s) | 301.1 ± 48.2 | 332 ± 60.89 | ns |
| Ao diameter (mm) | 0.59 ± 0.04 | 0.52 ± 0.02 | ns |
| LVSV (µL) | 9.93 ± 2.8 | 3.96 ± 0.53 | 0.033* |

Vent., ventricles; Vent/Body represents the ratio ventricles (without atria) and body; Pa, Pulmonary artery peak velocity; Ao, Aortic internal diameter was obtained at end-systole; LVSV, Left Ventricular Stroke Volume. Statistically significant result was considered when **P*>0.05. ns, means no statistical significant results. Data are means ± standard error of the mean (SEM).
